# Supplementary material for: High Rate of Mutational Events in SARS-CoV-2 Genomes across Brazilian Geographical Regions, February 2020 to June 2021
Source: Viruses. 2021 Sep 10;13(9):1806. doi: 10.3390/v13091806 (PMC8473193; doi:10.3390/v13091806)
Supplement: Supplementary file 1 [file viruses-13-01806-s001.zip › viruses-1344495-supplementary.pdf]

Supplementary Materials

High Rate of Mutational Events in SARS-CoV-2 Genomes across Brazilian Geographical Regions, February 2020 to June 2021

Ueric José Borges de Souza [1,†], Raíssa Nunes dos Santos [1,†], Fabrício Souza Campos [1], Karine Lima Lourenço [2], Flavio Guimarães da Fonseca [2], Fernando Rosado Spilki [3,*] and Corona-ômica.BR/MCTI Network [4]

[1] Laboratório de Bioinformática e Biotecnologia, Campus de Gurupi, Universidade Federal do Tocantins, Gurupi, 77402-970, Brazil; uericjose@gmail.com (U.J.B.d.S.); engraissanunes@gmail.com (R.N.d.S.); camposvet@gmail.com (F.S.C.)

[2] Laboratório de Virologia Básica e Aplicada, Departamento de Microbiologia, Instituto de Ciências Biológicas, Universidade Federal de Minas Gerais, Belo Horizonte, 31270-901, Brazil; karine_lourenco@hotmail.com (K.L.L.); fdafonseca@icb.ufmg.br (F.G.d.F.)

[3] Laboratório de Saúde Única, Feevale Techpark, Universidade Feevale, Av. Edgar Hoffmeister, 600, Zona Industrial Norte, Campo Bom, 93700-000, Brazil

[4] Laboratório de Microbiologia Molecular, Universidade Feevale, Rodovia ERS-239, 2755, Prédio Vermelho, Piso 1, sala 103, Vila Nova, Novo Hamburgo, 93525075, Brazil

* Correspondence: fernandors@feevale.br

† Both authors contributed equally.

Table S1. Region, total of genomes, and Ln function.

Region	n Genomes	nG/ΣG	ln(nG/ ΣG)	N Lineages	nL/ΣL	ΣG/n Maximum Lineages	ln(ΣG/ΣL)	n Max Lineages
South America	26,257	0.01596517662	−4.13734539	439	0.0489682097	138.9259259	4.933940884	189
Oceania	15,484	0.00941481486	−4.665470778	362	0.04037925265	62.18473896	4.130109615	249
Europe	1,000,285	0.6082083517	−0.497237772	4738	0.5284997211	1046.323222	6.953037604	956
Asia	112,901	0.0686477665	−2.678766682	1552	0.1731176799	220.5097656	5.395941982	512
North America	477,842	0.29054469	−1.285420251	1270	0.141662019	652.7896175	6.481254899	649
Africa	11,873	0.00721920028	−4.931011096	604	0.06737311768	49.67782427	3.905558642	239
**ΣG**	**1,644,642**	**-**	**-**	**8965**	-	-	-	-

Table S2. Estimated correction factor (0.06485746617).

Region	n Genomes	x fc	n Max Lineages	x fc	G/L	n G/fcL
South America	26,257	1702.962489	189	19.66949247	86.58	1334.91
Oceania	15,484	1004.253006	249	25.91377579	38.75	597.52
Europe	1,000,285	64,875.95055	956	99.49224763	652.07	10,053.90
Asia	112,901	7322.472789	512	53.28455103	137.42	2118.83
North America	477,842	30,991.62135	649	67.54233129	458.85	7074.70
Africa	11,873	770.0526959	239	24.87306191	30.96	477.34

Table S3. Pango lineages and number of SARS-CoV-2 genomes across Brazilian States.

States	Number of Genomes	Lineages
Acre	57	B.1, B.1.1, B.1.1.28, B.1.1.33, B.1.1.348, B.1.212, N.9, P.1 and P.2
Alagoas	289	B.1.1, B.1.1.28, B.1.1.33, B.1.1.372, N.9, P.1, P.1.1, P.1.2 and P.2
Amapá	91	B.1.1.28, B.1.1.33, B.1.160.25, P.1 and P.2
Amazonas	336	A.2, B.1.1, B.1.1.28, B.1.1.33, B.1.1.378, B.1.111, B.1.195, B.1.212, B.1.258, N.1, N.9, P.1 and P.2
Bahia	430	A.1, B.1, B.1.1, B.1.1.28, B.1.1.33, B.1.1.378, B.1.1.7, B.1.525, B.3, C.14, N.9, P.1, P.1.2 and P.2
Ceará	122	B.1.1, B.1.1.28, B.1.1.33, B.1.1.332, B.1.212, N.9, P.1 and P.2
Distrito Federal	28	B, B.1.1, B.1.1.28, B.1.1.33, B.1.1.7, B.1.177.32, P.1 and P.2
Espírito Santo	185	B.1.1, B.1.1.28, B.1.1.33, B.1.1.7, B.40, N.9, P.1, P.1.2 and P.2
Goiás	803	B.1.1, B.1.1.28, B.1.1.33, B.1.1.332, B.1.1.7, B.1.617.2, N.9, P.1, P.1.1, P.1.2 and P.2
Maranhão	149	B.1.1, B.1.1.28, B.1.1.33, B.1.1.332, B.1.234, B.1.617.2, N.10, N.9, P.1, P.1.2 and P.2
Mato Grosso	4	B.1.1.28, B.1.1.33 and B.1.1.7
Mato Grosso do Sul	104	B.1, B.1.1, B.1.1.28, B.1.1.33, B.1.1.7, B.1.212, B.1.240, B.1.547, N.4, P.1 and P.2
Minas Gerais	315	B.1, B.1.1, B.1.1.28, B.1.1.33, B.1.1.7, B.1.2, B.1.212, B.1.234, B.1.617.2, B.1.9, P.1, P.1.1, P.1.2 and P.2

Pará	304	B.1, B.1.1, B.1.1.28, B.1.1.33, B.1.1.378, B.1.212, B.39, N.9, P.1, P.1.2 and P.2
Paraíba	238	B.1.1, B.1.1.28, B.1.1.33, B.1.1.462, B.1.1.7, B.1.212, B.1.524, N.9, P.1, P.1.2 and P.2
Paraná	362	B.1, B.1.1, B.1.1.28, B.1.1.33, B.1.1.7, B.1.195, B.1.617.2, N.9, P.1, P.1.2 and P.2
Pernambuco	117	B, B.1, B.1.1, B.1.1.117, B.1.1.192, B.1.1.28, B.1.1.33, B.1.1.74, B.1.212, N.9, P.1 and P.2
Piauí	18	B.1.1.28, B.1.1.33, B.1.524, N.9, P.1 and P.2
Rio de Janeiro	3114	A.2, B.1, B.1.1, B.1.1.277, B.1.1.28, B.1.1.33, B.1.1.348, B.1.1.519, B.1.1.7, B.1.1.97, B.1.111, B.1.617.2, B.39, N.1, N.4, N.9, P.1, P.1.1, P.1.2, P.2 and P.4
Rio Grande do Norte	181	B.1, B.1.1, B.1.1.28, B.1.1.33, B.1.1.74, B.1.212, N.1, N.9, P.1 and P.2
Rio Grande do Sul	1049	B, B.1, B.1.1, B.1.1.12, B.1.1.161, B.1.1.28, B.1.1.33, B.1.1.332, B.1.1.7, B.1.195, B.1.575, B.1.91, N.1, P.1, P.1.1, P.1.2 and P.2
Rondônia	32	B.1.1.28, B.1.1.33, B.1.212, N.9, P.1 and P.2
Roraima	36	B.1, B.1.1.28, B.1.1.33, P.1, P.1.1 and P.2
Santa Catarina	502	B.1, B.1.1, B.1.1.28, B.1.1.33, B.1.1.332, B.1.1.7, B.1.195, B.6, C.36, N.9, P.1, P.1.2 and P.2
São Paulo	7719	B, B.1, B.1.1, B.1.1.28, B.1.1.318, B.1.1.33, B.1.1.332, B.1.1.393, B.1.1.519, B.1.1.523, B.1.1.7, B.1.177.52, B.1.195, B.1.221, B.1.234, B.1.351, B.1.617.2, B.3, C.37, N.6, N.9, P.1, P.1.1, P.1.2, P.2 and P.4
Sergipe	303	B.1, B.1.1, B.1.1.28, B.1.1.33, B.1.1.7, B.1.212, B.59, N.9, P.1, P.1.2 and P.2
Tocantins	65	B.1.1, B.1.1.28, B.1.1.33, P.1, P.1.1, P.1.2 and P.2
